# The Role of Gamma Oscillations During Integration of Metaphoric Gestures and Abstract Speech

**DOI:** 10.3389/fpsyg.2018.01348

**Published:** 2018-07-30

**Authors:** Yifei He, Arne Nagels, Matthias Schlesewsky, Benjamin Straube

**Affiliations:** ^1^Translational Neuroimaging Lab, Department of Psychiatry and Psychotherapy, Marburg Center for Mind, Brain and Behavior, Philipps-University Marburg, Marburg, Germany; ^2^Department of General Linguistics, Johannes Gutenberg University Mainz, Mainz, Germany; ^3^School of Psychology, Social Work and Social Policy, University of South Australia, Adelaide, SA, Australia

**Keywords:** gamma oscillations, metaphor, gesture, multisensory integration, figurative language

## Abstract

Metaphoric (MP) co-speech gestures are commonly used during daily communication. They communicate about abstract information by referring to gestures that are clearly concrete (e.g., raising a hand for “the level of the football game is high”). To understand MP co-speech gestures, a multisensory integration at semantic level is necessary between abstract speech and concrete gestures. While semantic gesture-speech integration has been extensively investigated using functional magnetic resonance imaging, evidence from electroencephalography (EEG) is rare. In the current study, we set out an EEG experiment, investigating the processing of MP vs. iconic (IC) co-speech gestures in different contexts, to reveal the oscillatory signature of MP gesture integration. German participants (*n* = 20) viewed video clips with an actor performing both types of gestures, accompanied by either comprehensible German or incomprehensible Russian (R) speech, or speaking German sentences without any gestures. Time-frequency analysis of the EEG data showed that, when gestures were accompanied by comprehensible German speech, MP gestures elicited decreased gamma band power (50–70 Hz) between 500 and 700 ms in the parietal electrodes when compared to IC gestures, and the source of this effect was localized to the right middle temporal gyrus. This difference is likely to reflect integration processes, as it was reduced in the R language and no-gesture conditions. Our findings provide the first empirical evidence suggesting the functional relationship between gamma band oscillations and higher-level semantic processes in a multisensory setting.

## Introduction

Metaphor is ubiquitous in daily communication. It is common to hear someone talking about the *ups* and *downs* of a day, and describing them as *bitter* or *sweet*. These constructions provide a means to communicate about abstract target representations by referring to source representations that are clearly more concrete (Lakoff and Johnson, 1980). Of note, the pervasive occurrence of metaphor is not restricted to verbal communications (Forceville and Urios-Aparisi, 2009). Non-verbal pictures and gestures can also be metaphoric (MP) (Cienki and Müller, 2008). For example, someone can complain about an excessively long lecture, not only by saying *the lecture goes on and on*, but also by adding a gesture, rolling his/her hand at the same time. To grasp the meaning of this co-speech gesture, a bridging between the concrete (literal) gesture and the abstract (MP) depiction of the endless *lecture* is necessary. Clearly, similar to that of linguistic metaphor, MP gestures also typically indicate or represent the source domain of a metaphor (McNeill, 1992). However, unlike linguistic metaphors, MP gesture is a multi-modal form in which the MP meaning is expressed or underpinned. In this sense, successful comprehension of MP co-speech gesture requires not only lower-level integration of audio–visual input, but also higher-level semantic processes, so that the construction of a coherent, and thus MP semantic representation is possible. Crucially, recent evidence from neurophysiology has emerged, suggesting that both processes may be related to de/synchronized brain oscillations in the gamma band (>30 Hz).

Gamma band oscillations are thought to play an important role during multisensory integration of not only lower-level and meaningless stimuli (Senkowski et al., 2005, 2007; Kaiser et al., 2006; Widmann et al., 2007), but also the formation of higher-level semantic presentations across modalities (Yuval-Greenberg and Deouell, 2007; Schneider et al., 2008; Diamond and Zhang, 2016; Drijvers et al., 2018). For instance, it has been suggested that increased gamma band power (40–50 Hz) supports cross-modal semantic matching when an auditory stimuli is primed with a congruent/incongruent picture (Schneider et al., 2008). Increased gamma band power (30–70 Hz) was also reported for congruent vs. incongruent visual and auditory stimuli (e.g., a cat picture with “Meow” vs. “Woof”) when they were presented simultaneously, thus suggesting the role of gamma oscillations during multimodal semantic integration. Diamond and Zhang (2016) also reported increased gamma band power (around 50 Hz) for emotionally congruent vs. incongruent face–speech combinations; however, no effects were observed for congruency at phonemic level, this finding thus lend further support to the role of gamma band oscillations during multimodal integration at semantic level. Additionally, a recent study from Drijvers et al. (2018) investigated gestural enhancement effect (bimodal co-speech gesture > speech-only) for in degraded speech and clear speech conditions, and they found more pronounced gamma band power increase (65–80 Hz) for the degraded speech conditions. Altogether, gamma band oscillations may be highly relevant to multisensory integration at a semantic level.

Interestingly, psycholinguistic research focusing at the oscillatory aspects of sentence processing suggests that there is also a close functional link between gamma-band oscillations and semantic unification/integration at the sentence level (Lewis and Bastiaansen, 2015). This line of research, similar to multisensory semantic integration, reported enhanced gamma band (ranging between 40 and 50 Hz) activity for semantically coherent and predictable sentences (Hald et al., 2006; Wang et al., 2012; Bastiaansen and Hagoort, 2015). Of note, the sensitivity of gamma oscillations is not restricted to semantic congruency. Nieuwland and Martin (2017) showed that referentially coherent vs. incoherent sentence expressions elicited increased gamma band power (40–80 Hz), and this effect is independent of modality, language, and the types of referential expression. More relevant to the current study, by comparing idioms with literal sentences, Rommers et al. (2013) found reduced gamma power (50–70 Hz) for idioms when compared with their literal counterparts, which may suggest that gamma oscillations are not only sensitive to semantic congruency, but also semantic complexity. It has to be noted that although these studies have unanimously reported gamma band effect for differential level of semantic/pragmatic processing, the exact spectral range among studies is not homogeneous. However, these gamma band effects all fall into the lower- and middle-gamma range (from 30 to 80 Hz), which is proposed, according to a recent theoretical framework by Lewis and Bastiaansen (2015), to support the match (integration) between highly predictable (and so pre-activated) lexical representations and the incoming linguistic input. This range characterizes itself from the higher gamma range (above 80 Hz), which is related to prediction errors (Giraud and Poeppel, 2012; Fontolan et al., 2014). Nevertheless, in sum, recent research indicates that gamma band oscillations (30–80 Hz) may support higher-level semantic processes during multisensory integration.

In the current electroencephalography (EEG) study, we directly tested the hypothesis that gamma band oscillations are specifically related to the neural integration of MP gestures and corresponding abstract speech. Importantly, this integrative process links a concrete (literal) visual input to an abstract (figurative) speech context, and is thus multisensory and semantic in nature. Departing from previous M/EEG studies that adopting the “mismatch” paradigm (comparing congruent vs. incongruent multimodal stimuli), in which the integration process may be confounded by conflict resolution (c.f., Green et al., 2009; Holle et al., 2010), we opted to the “additive” paradigm where effects in unimodal conditions (visual-only and auditory-only) were compared to the bimodal condition. This approach of examining multisensory integration has been proven to be highly successful in functional magnetic resonance imaging (fMRI) studies (Beauchamp et al., 2004; Calvert and Thesen, 2004; He et al., 2015, 2018), and has recently been adopted to M/EEG research with a focus on brain oscillations (He et al., 2015, 2018; Drijvers et al., 2018). Therefore, we compared the processing of MP co-speech gestures (MPG) with the processing of iconic co-speech gestures (ICG) that semantically integrate concrete (literal) gesture and concrete (literal) speech, which can be semantically less complex. Additionally, we compared the MP vs. IC gestures under various semantically uni-modal control conditions where multimodal semantic integration does not happen (with semantically meaningful input in either visual-only or auditory-only modality, see the “Stimuli” section). We thus hypothesized that the integration difference between MPG vs. ICG will elicit power decrease in the gamma band. Moreover, we hypothesized that this effect will be source-localized to the left inferior frontal gyrus (left IFG), because it has been argued that the left IFG is sensitive to semantic complexity that is related to the processing of linguistic metaphor (Rapp et al., 2004, 2007; Eviatar and Just, 2006). Additionally, recent evidence from fMRI suggests that the left IFG is involved in the multisensory integration of MPG (Kircher et al., 2009; Straube et al., 2011).

## Materials and Methods

### Participants

Twenty-two participants (14 female, mean age = 24.02, range 21–32) participated in this experiment and they were paid 15 euros for the participation. All participants were right-handed as assessed by a questionnaire on handedness (Oldfield, 1971). They were all native German speakers and had no Russian (R) proficiency. None of the participants reported any hearing or visual deficits. Exclusion criteria were history of relevant medical or psychiatric illnesses of the participants. All subjects gave written informed consent prior to participation in the experiment. The study was approved by the local ethics committee. The data from two participants were discarded because of excessive artifacts during recording.

### Stimuli

**FIGURE 1:**
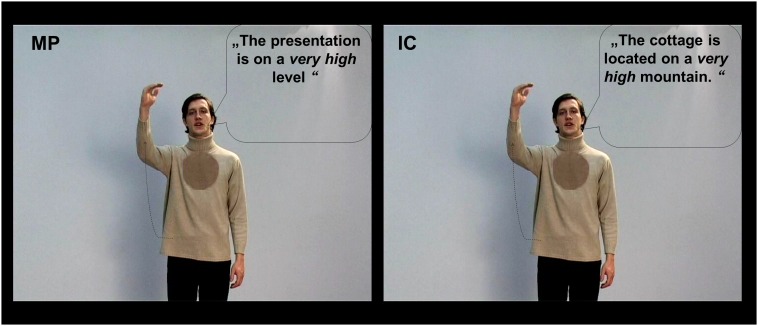
Picture illustration for the metaphoric (MP) and iconic (IC) gesture conditions in the co-speech condition (G). Same videos were also presented with two additional modality conditions: gestures with foreign Russian (R) sentences, German sentences without any gestures (N). For illustrative purposes the spoken German sentences were translated into English. For this figure all spoken sentences are written into speech bubbles.

The participants were shown video clips of co-speech gestures selected from a large pool of videos (Green et al., 2009; Kircher et al., 2009; Straube et al., 2011). All videos lasted 5 s and contained only one simple subject–verb–object sentence spoken by an actor. The actor was a highly proficient German–R early-bilingual speaker. He performed two types of co-speech gestures: these were MP gestures and IC gestures. The gestures in the MP condition illustrate the form, size, or movement of an abstract concept that is mentioned in the associated speech; whereas the gestures in the IC condition illustrate the form, size, or movement of something concrete, as conveyed by the associated sentence (McNeill, 1992, 2008). For both gesture types, videos of three modalities were constructed and were shown to the participants: besides the co-speech condition (G) that shows both gestures and German sentences, we included two additional control conditions, these were (1) gestures with incomprehensible R sentences and (2) German sentences with no gestures (N). Importantly, although the videos in both the co-speech (G) and the N conditions are easily understandable, the videos in the R condition can be difficult to interpret: for both MP and IC gestures, their interpretation depends heavily on the co-occurring speech (McNeill, 2008), which is not accessible for our German participants in the R condition. Thus, the R condition can be considered as a non-semantic visual–auditory control condition to the multimodal semantic co-speech (G) condition, and the N condition serves as a semantic auditory-only control condition. Overall, we used 576 videos (32 videos per condition × 6 conditions × 3 sets). Each participant only watched one set of stimuli. Additionally, for each participant, 32 filler videos containing R sentences with meaningless gestures were also presented. For an illustration of the videos, please refer to **Figure 1**.

### Stimulus Ratings

Twenty additional native German-speaking participants, who did not take part in the EEG experiment, rated the stimulus videos on understandability, naturalness, and concreteness on a scale from 1 to 7 (1 = very low to 7 = very high). Rating results and further timing parameters (speech duration and gesture duration) for the six conditions are listed in **Table 1**. Analyses of variance with two factors (GESTURE and MODALITY) were performed for the all stimulus and rating parameters between the conditions.

**Table 1:** Mean stimulus durations and rating parameters for videos in the six experimental conditions (SD: standard deviation).

	Understandability		Naturalness		Concreteness		Speech duration		Gesture duration	
					
	Mean	*SD*	Mean	*SD*	Mean	*SD*	Mean	*SD*	Mean	*SD*
MPG	6.76	0.18	4.69	0.60	4.48	0.53	2.51	0.40	2.40	0.37
ICG	6.8	0.14	4.96	0.60	6.03	0.44	2.37	0.32	2.70	0.48
MPR	2.06	0.6	4.26	0.34	2.87	0.38	2.49	0.43	2.37	0.41
ICR	2.37	0.73	3.88	0.53	3.29	0.76	2.32	0.48	2.67	0.45
MPN	6.54	0.75	3.92	0.34	2.99	0.47	2.44	0.37	N/A	N/A
ICN	6.58	0.18	3.58	0.34	4.33	0.32	2.33	0.35	N/A	N/A


For understandability, there was significant main effect of both GESTURE [*F*_(1,95)_ = 12.07, *p* < 0.001] and MODALITY [*F*_(2,190)_ = 4787.07, *p* < 0.001]. There was also a significant interaction between GESTURE ^∗^ MODALITY [*F*_(2,190)_ = 4.19, *p* < 0.03]. Resolving this interaction by MODALITY, simple effect *t*-tests showed significant difference for GESTURE only in the R condition [*t*_(95)_ = 3.21, *p* < 0.002]. The ratings for understandability clearly suggest that there was no general difference between MP vs. IC gestures in terms of understandability. However in the R condition, IC gestures were easier to understand than MP gestures, even if both types in this modality were rated low in terms of understandability.

For naturalness, we observed a significant main effect of GESTURE [*F*_(1,95)_ = 17.88, *p* < 0.001] and MODALITY [*F*_(2,190)_ = 237.70, *p* < 0.001]. There was also a significant interaction of GESTURE ^∗^ MODALITY [*F*_(2,190)_ = 26.53, *p* < 0.001]. For simple effect *t*-tests, we then compared MP vs. IC gestures within each modality. We observed significant lower naturalness ratings for the MP gestures in the co-speech (G) condition [*t*_(95)_ = 3.18.10, *p* < 0.002], however, in both the R and the N conditions, MP gestures were rated as more natural [*t*_(95)min_ = 5.92, *p*_max_ < 0.001].

With regard to concreteness, we observed a significant main effect of GESTURE [*F*_(1,95)_ = 1004.49, *p* < 0.001], together with a significant main effect of MODALITY [*F*_(2,190)_ = 811.96, *p* < 0.001]. There was also a significant GESTURE ^∗^ MODALITY interaction [*F*_(1,95)_ = 68.37, *p* < 0.001]. Simple effect *t*-tests suggested that, within all modalities, MP gestures were less concrete than IC gestures [*t*_(95)__min_ = 5.09, *p*_max_ < 0.001]. The concreteness ratings clearly suggest that MP gestures were less concrete than that of IC gestures.

For stimulus durations, speech duration was found to be longer for MP gestures, as revealed by a significant main effect of GESTURE [*F*_(1,95)_ = 17.60, *p* < 0.001]. However, in terms of gesture duration, for all four conditions with gestures, MP gestures were generally shorter than IC gestures, as revealed by a significant main effect of GESTURE [*F*_(1,95)_ = 62.08, *p* < 0.001].

### Experimental Procedure

After participants gave written informed consent, an EEG-cap (EasyCap GmbH, Herrsching, Germany) was fastened to the participant’s head and the electrodes were attached at their according sites. Abrasive electrode gel was administered and care was taken to keep all impedances below 5 kΩ. Participants were comfortably seated in a sound-proof room in front of a standard 19^″^ TFT monitor with a distance of approximately 70 cm. Stimuli were presented using the Presentation software package (Neurobehavioral Systems, Inc., Albany, CA, United States). Auditory stimuli were presented using a pair of stereo speakers and the loudness of the speaker was kept constant across participants. For each participant, an experimental session comprised 224 videos (32 for each condition and 32 additional filler videos) and consisted of two 16-min blocks. Each block contained 112 trials with a matched number of items from each condition (16) and 16 filler trials. The stimuli were presented in pseudo-randomized order and were counterbalanced across participants. Each video clip was followed by a gray background with a variable duration of 2154–5846 ms (jitter average: 4000 ms). Participants were instructed to watch the videos and to respond each time they saw a new video and by pressing a button in a joystick with the index finger. This was done to ensure that they paid attention during all videos. This implicit-encoding task was chosen to focus participants’ attention to the videos and enabled us to investigate implicit speech and gesture processing. Before EEG data collection, each participant participated in at least 10 practice trials that were different from those used in the experiment.

### EEG Recording

Electroencephalography was collected from 64 sintered Ag/AgCl Electrodes attached to the EasyCap (EasyCap GmbH, Herrsching, Germany) according to the international 10–10 System. The reference electrode was located at the vertex between Fz and Cz and the ground electrode was located at the forehead in front of Fz. All input impedances were kept below 5 kΩ. Additionally, the vertical electrooculogram (VEOG) was recorded from one electrode located underneath the left eye. The “Brain Amp” (Brain Products) amplifier was used to sample data at 500 Hz with a resolution of 0.1 μV. Trigger signals from stimulus and participants responses were acquired together with the EEG using the Brain Vision Recorder software (Brain Products GmbH, Munich, Germany).

### EEG Data Analysis

All analyses were carried out using the Brain Vision Analyzer 2.1 (Brain Products GmbH, Munich, Germany) and the Fieldtrip toolbox for EEG/MEG analysis (Maris and Oostenveld, 2007). Data were firstly high-pass filtered at 0.1 Hz and low-pass filtered at 125 Hz, and then re-referenced to the average of the all electrodes. EOG and muscle artifacts were identified and rejected via an infomax independent component analysis with maximum of two EOG-related ICs rejected for each participant. Then the raw EEG was segmented into -0.5–1.5 s segments around the onset of each critical word (the word during which the meaning of gesture and speech coincides, such as good in the sentence “the actor did a good job”). The onset of the critical word is considered to be most important for the temporal synchronization between speech and gesture (Habets et al., 2011; Obermeier et al., 2011; Obermeier and Gunter, 2014), and it is chosen as the time point for ERP studies targeting at gesture-speech integration (Kelly et al., 2004; Wu and Coulson, 2005, 2010; Özyürek et al., 2007). Similar onsets were also chosen for the investigation of gesture-speech integration for other types of gestures (e.g., emblems and speech) using EEG, fMRI, and simultaneous EEG–fMRI (He et al., 2015, 2018). The onsets were similarly defined for the two unimodal conditions.

After the segmentation, additional muscle artifacts and slow drifts were automatically detected and rejected based on the amplitude distribution across trials and channels (as implemented in Fieldtrip toolbox). Cutoffs for these artifacts were set at *z* = 20. On average, 5.04 out of 32 trials were rejected for each condition, with no significant difference between conditions. In order to reveal event-related power oscillations of the EEG, time-frequency representations (TFRs) of the single trial data were computed. TFRs were computed in two different frequency ranges to optimize the trade-off between time and frequency resolution. In the low frequency range (2–30 Hz) a constant Hanning taper of 400 ms was used to compute power changes in frequency steps of 1 Hz and time steps of 0.05 s; in the high frequency range (30–80 Hz), the time-frequency analysis was carried out using a multi-taper approach with frequency steps of 2.5 Hz and time steps of 0.05 s (Mitra and Pesaran, 1999). All TFRs were interpreted based on baseline corrected (-0.5 to -0.15 s) relative power changes.

For statistical analyses, we firstly used a cluster-based random permutation test (Maris and Oostenveld, 2007), as this approach naturally handles multiple-comparisons problem. This approach was used to evaluate pair-wise comparisons between MP and IC G, R, and the N conditions, respectively. The procedure of the statistical analysis is briefly described in the following: firstly, for every data point (time-frequency-channel point), a simple dependent-samples *t*-test is performed which results in uncorrected *p*-values. Secondly, all significant data points (*p* < 0.05) are grouped as clusters (here: clusters of at least five neighboring electrodes, with maximum distance of 2.5 cm). For each cluster the sum of the *t*-statistics is used in the cluster-level statistics. Finally, a Monte-Carlo non-parametrical permutation method with 1000 repetitions is implemented to estimate type I-error controlled cluster significance probabilities (*p* < 0.025) in the time-frequency-channel space. In a second step, to test potential interaction effects between our experimental factors GESTURE and MODALITY, we run a repeated-measures ANOVA for all conditions based on the averaged power within the significant time-frequency-electrode space.

### EEG Source Localization

Source localization is able to provide anatomical insights into the EEG effects of interest. We investigated the neural generators of the observed responses using source localization to provide a tentative link to neuroimaging studies of metaphors. A frequency-domain adaptive spatial filtering imaging of coherent sources (DICS) algorithm (Gross et al., 2001), as implemented in the Fieldtrip toolbox for EEG/MEG analysis (Oostenveld et al., 2011), was carried out. Source analysis was performed for the time-frequency windows in the gamma band in which significant results were obtained on the scalp level (see the “Results” section). Firstly, participants’ individual electrode positions were warped to the cortical mesh of a standard Boundary Element head model. The forward model was computed on an 8 mm grid of source positions covering the whole brain compartment of the BEM. For source analysis, common space filters were constructed using the leadfield of each grid point and the cross-spectral density matrix (CSD). The CSD matrix was computed based on an additional time-frequency analyses for data segments of interests and their respective baseline. The source activity volumes were corrected during the baseline period, and then were compared by means of paired *t*-tests. This procedure resulted in a source-level *t*-statistics of gamma power change for each voxel in the volume grid. Lastly, the *t*-statistics were passed to a cluster-based permutation test at the voxel level for correction of multiple comparisons.

### The Relationship Between Stimulus Features and Gamma Band Power

Based on our results in the gamma band (see the “Results” section), we sought to further explore how gamma band power were related to stimulus parameters (in terms of speech and gesture durations) as well as rating measurements for both types of gestures in different modalities. To this end, we used mixed-effects modeling with crossed random-effect factors for subjects and items (Baayen et al., 2008), so as to analyze the relationship between log-transformed gamma band power and each individual stimulus-related features (speech duration, gesture duration, concreteness ratings, naturalness ratings, and understandability ratings). For each stimulus feature, we started from simpler mixed-effects models with subject and items as random factors that include modality, gesture-type, and the stimulus feature as predictors, and then looked at whether interactions between these factors improved the model fit. However, for all stimulus features, no significant effects were observed when they were modeled as fixed effects.

## Results

### Time-Frequency Results

**FIGURE 2:**
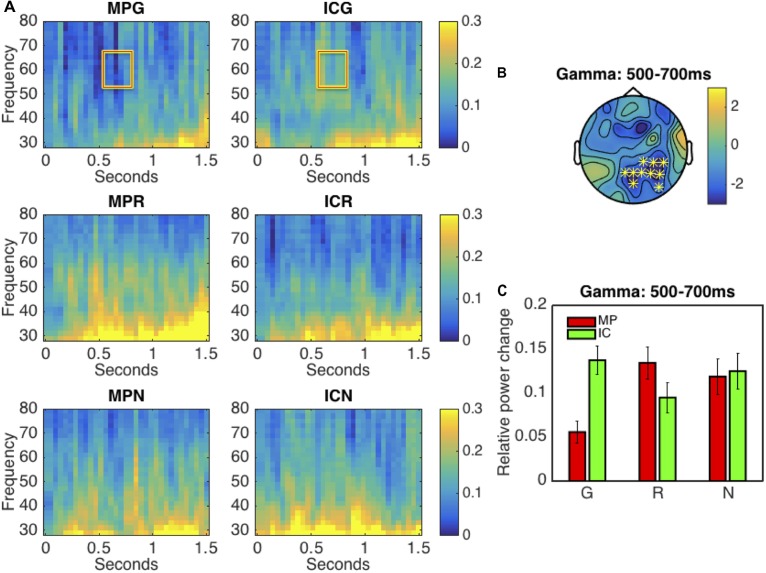
Results of the time-frequency (TF) analysis. **(A)** The panel shows the TF representations of all conditions at electrode P4. **(B)** This panel illustrates the scalp distribution of the significant cluster in the gamma band (50–70 Hz) in the 500–700 ms interval. The electrodes forming the significant cluster are marked with asterisks on the topographic plot. Panel **(C)** shows the averaged (across trials and subjects) relative power changes in the gamma band (50–70 Hz) for all experimental conditions in the 0.5–0.7 s time interval at all electrodes in the significant cluster (as in panel **B**).

Firstly, we directly compared the oscillatory differences between MP and IC gestures within all modality conditions. In the low frequency range (2–30 Hz), all comparisons revealed no significant effect. In the high frequency range (30–80 Hz), in the G condition, we observed significant power decrease (*p* < 0.005) in the gamma band (50–70 Hz) for the MP gestures when compared to IC gestures. This effect elapsed between 500 and 700 ms, and had a parietal scalp distribution, as illustrated in **Figures 2A–C**. However, in both MP vs. IC comparisons within the R and N conditions, no significant effects were observed. Results from the repeated-measures ANOVA in the gamma band power suggested the specificity of the co-speech modality condition: we observed a significant interaction between factors GESTURE and MODALITY [*F*_(2,38)_ = 4.57, *p* < 0.02], but no main effect of either GESTURE or MODALITY. Resolving the interaction by MODALITY, simple effect *t*-test showed only significant effect of GESTURE in G condition [*t*_(19)_ = 3.82, *p* < 0.002], but not in the other control conditions [*t*_(19)max_ = 1.70, *p*_min_ = 0.11]. Resolving the interaction by factor GESTURE, we observed no significant difference between all IC gestures [*F*_(2,38)_ = 1.18, *p* < 0.31]. Between all MP gestures, we observed a significant difference [*F*_(2,38)_ = 4.97, *p* < 0.012]. Simple effect *t*-test with Bonferroni correction showed that there was a significant difference between MPG vs. MPR [*t*_(19)_ = 3.47, *p* < 0.005] and MPG vs. MPN [*t*_(19)_ = 2.52, *p* < 0.04], however, no difference was observed for MPR vs. MPN [*t*_(19)_ = 0.50, *p* < 0.61]. This interaction is illustrated in **Figure 2C**. The results were in line with our hypothesis that gamma band oscillations may support the semantic integration of MP vs. ICG.

### Source Localization Results

**FIGURE 3:**
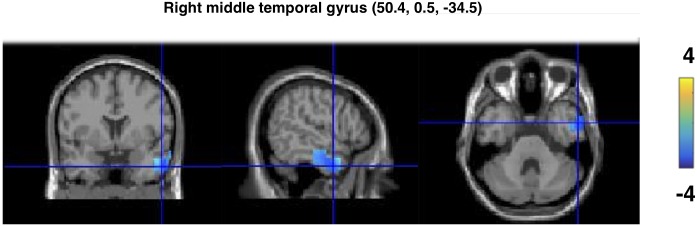
Source reconstruction results of the gamma band effect for the MP vs. IC gesture in the co-speech condition. The sources were spatially normalized to the MNI template brain. The peak of the source is located in the right middle temporal gyrus.

Source localization on the significant cluster for the gamma band effect for the MPG vs. ICG comparison yielded a peak maxima in the right middle temporal gyrus (*x* = 54.5, *y* = 0.5, *z* = -35.5). The peaks were observed in a cluster including both the right middle temporal gyrus and the right inferior temporal gyrus, as illustrated in **Figure 3**.

## Discussion

In the current study, we tested whether gamma oscillations play a specific role in the integration of MP gestures and corresponding abstract speech. We found that, in the co-speech gesture condition, MP gestures yielded decreased gamma power when compared with IC gestures (MPG vs. ICG), and this effect was source localized to the right middle temporal gyrus. Moreover, this effect was specific to the co-speech modality. Thus, gamma power oscillations may specifically support the integration of MPG.

Previous studies on multisensory integration of both lower- and higher-level stimuli found gamma band power decrease for incongruent vs. congruent audio–visual stimuli, thus suggesting the relevance between gamma oscillations and multisensory integration (Yuval-Greenberg and Deouell, 2007; Schneider et al., 2008; Diamond and Zhang, 2016). As we did not directly apply the mismatch paradigm in the current study, our findings may not be directly comparable in terms of research design. However, previous literature suggests that the effect of multisensory integration can be obtained by not only the incongruent vs. congruent comparison, but also the comparisons between Bimodal > Audial and Bimodal > Visual modalities (Calvert and Thesen, 2004; Green et al., 2009; Holle et al., 2010). More importantly, this paradigm has also been applied to recent EEG studies that directly investigated the integration between gesture and speech (Biau and Soto-Faraco, 2013; Biau et al., 2015; He et al., 2015; Drijvers et al., 2018) and fMRI studies focusing on integration of MP gestures (Kircher et al., 2009; Straube et al., 2011). In the current study, by carrying out a similar paradigm, we observed that the gamma band power may reflect the integration between MP gestures and corresponding abstract speech, as it differs between the co-speech MPG condition and both semantically unimodal conditions (MPG vs. MPR and MPG vs. MPN). More importantly, the gamma band power was not sensitive to comparisons among the three IC gesture conditions. Additionally, as we observed significant difference between MPG and ICG conditions, as a result, even if a comparison between MPG vs. ICG does not speak for multisensory integration *per se*, such comparison may represent the differential level of semantic complexity (MP vs. literal) during multisensory integration of two gesture types. Of note, gamma band effect on lower-level multisensory integration commonly peaks as early as before 100 ms (Murray et al., 2002; Senkowski et al., 2005); for both studies reporting gamma band effect for semantic picture of mismatch and sound (Yuval-Greenberg and Deouell, 2007; Schneider et al., 2008), the peak latency was around 300 ms. In the current study, our effect was clearly later (500–700 ms) and is more comparable gamma band effects in sentence processing studies in terms of latency (Wang et al., 2012; Rommers et al., 2013; Nieuwland and Martin, 2017). It is also comparable to the gamma band effect from a recent study from Drijvers et al. (2018), which directly investigates the gestural enhancement effect (comparing directly between audio–visual and visual-only conditions), which ranges between 300 and 900 ms after the critical word onset. Thus, this latency difference between studies suggests that gamma oscillations may potentially support lower- and higher-level multisensory integration at different stages, and in the current study, it is more likely to be related to higher-level semantic integration between gesture and speech.

The gamma band difference between the MP and ICG can be examined under recent proposals from psycho/neurolinguistic literature concerning the role of gamma band oscillations during sentence level processing (Bastiaansen and Hagoort, 2015; Lewis and Bastiaansen, 2015). It has been reported that, during sentence processing, when the semantic representation of a newly encountered word matches its predicted semantic feature from the preceding context, the gamma band synchronizes; this effect has been observed for not only highly predicable words and congruent words within a sentences (Hald et al., 2006; Wang et al., 2012; Rommers et al., 2013; Bastiaansen and Hagoort, 2015), but also for referentially more coherent sentences (Nieuwland and Martin, 2017). Thus, it has been proposed that gamma oscillations, especially the lower- and middle-gamma oscillations (30–80 Hz) reflect an interaction between top-down prediction and bottom-up checking for semantic features (Lewis and Bastiaansen, 2015). Importantly, according to this account, whenever there is no difference in the level of semantic prediction, there should be no difference in the gamma band power. However, the gamma band effect in the current study may not be exactly derived from this mechanism: in both semantically unimodal conditions, we observed reduced gamma band effects by comparing MP and IC gestures in either gesture-only (R) or N modalities, even if there could be a differential degree of semantic complexity originated from the MP vs. literal comparison, especially in the N condition. This is clear indication that at the critical word of integration, there may be comparable degree of prediction based on either the auditory-speech or visual-gesture. Bearing this into consideration, the observed effect in the co-speech MPG vs. ICG comparison may not support the predictive, but the cross-modal integrative nature of the gamma effect.

We thus argue that our gamma band findings are mostly compatible with the integration/semantic unification account (Bastiaansen and Hagoort, 2015), even though this account may need to be extended to a multisensory environment. In the case of multisensory integration, gamma band oscillations may represent the semantic integration across modalities. In our case, this integration may differ between (1) integrating a concrete gesture with an abstract sentence (in MP gestures) and (2) integrating a concrete gesture with a concrete sentence (in IC gestures), and the higher degree of semantic complexity for the former integration process leads to modulated gamma power. This account is able to derive the gamma band findings from Yuval-Greenberg and Deouell (2007): as they only looked at simultaneously presented picture and sound, a prediction that is similar to the sentence processing can hardly occur. Therefore, the increased gamma power for congruent picture and sound can be best explained by semantic integration across modalities. The account can also be corroborated by two recent studies that examined compositional aspects of sentence processing (Fedorenko et al., 2016; Lam et al., 2016). Using electrocorticography (ECoG), Fedorenko et al. (2016) found increased gamma power for the comprehension of normal sentences when compared with both word lists and sentences without meaning, and concluded that decreased power in the gamma band reflects the increasing complexity of the evolving semantic representation when a sentence unfolds. In a similar vein, an MEG study comparing sentences with scrambled word lists also showed decreased gamma band power for word lists (Lam et al., 2016). Along this argument, the gamma band effect in the current study, as well as during the comparison between idioms and literal words can be easily accounted for (Rommers et al., 2013): in both studies, the semantic complexity between the literal condition and the more figurative conditions (idioms and MP gestures) were clearly greater, thus lead to decreased power in the gamma band.

It has to be noted that even if the observed effects in the gamma band may be related to multimodal semantic integration for complex semantic features, as in the case of MP co-speech gestures, we were unable to identify a clear relationship between single-trial gamma power and stimulus features including speech and gesture durations and ratings scores on concreteness, naturalness, and understandability. There are a number of explanations: firstly, even if these stimulus features provide information on both lower- and higher-order characteristics, none of these features directly address the critical word. This is however the position that directly relates to semantic integration between gesture and speech (Obermeier and Gunter, 2014; He et al., 2018), and was analyzed to reveal the gamma-band effect. As a result, the obtained stimulus features were probably not sensitive enough to predict gamma-band power on a single-item basis. Future experiments maybe considered to systematically test the relationship between gamma band power and trial-based measures that may directly reflect semantic integration between gesture and speech [e.g., behavioral performance for the semantic-probe task, as in Kelly et al. (2010) and Zhao et al. (2018), these measures are not available for the current study]. The lack of clear relationship between gamma power and stimulus features may also be considered as evidence that gamma oscillations in general supports higher-order semantic processes (Yuval-Greenberg and Deouell, 2007; Lewis and Bastiaansen, 2015; Fedorenko et al., 2016; Drijvers et al., 2018), which maybe functionally dissociable from oscillations in the lower frequency ranges such as the theta band that are more often reported to be related to lower-order processes [Luo et al. (2010) and Giraud and Poeppel (2012), but see Gross et al. (2013) and Di Liberto et al. (2015) for the role of gamma oscillations in auditory perception]. Nevertheless, this functional dissociation requires further fine-grained experiments that look at both lower- and higher-order processes, and possibly their interactions, within an experimental paradigm.

The source of our gamma band effect between the MPG vs. ICG comparison was localized to the right middle temporal gyrus. This finding contradicts our hypothesis that the left IFG may support the semantic integration of MP vs. IC gestures, which was predominantly based on fMRI findings (Rapp et al., 2004; Eviatar and Just, 2006; Rapp et al., 2007). It has long been debated that whether the right hemisphere (RH) plays a selective role during the processing of non-literal language, especially with regard to linguistic metaphors (Mashal et al., 2005, 2007; Rapp et al., 2007; Cardillo et al., 2012; Lai et al., 2015). Importantly, even if the right fronto-temporal regions have been classically linked to the processing of figurative language (Kiehl et al., 1999; Kircher et al., 2001), with regard to the processing of MP vs. literal speech, most studies reported either predominantly increased activation in the left IFG (Rapp et al., 2004; Eviatar and Just, 2006; Lee and Dapretto, 2006; Rapp et al., 2007), or bilateral activation within both the left IFG and the right fronto-temporal regions (Bottini et al., 1994; Mashal et al., 2005, 2007; Cardillo et al., 2012; Lai et al., 2015). Despite the diversity in data collection methods (PET, fMRI) and research design, a consensus of these studies seems to suggest that, while the RH is not selectively involved in the processing of metaphor *per se*, it may support the processing of differential level of familiarity and novelty that are involved in metaphors (Mashal et al., 2007; Cardillo et al., 2012; Lai et al., 2015). In the current study, we found potential correlational relationship between concreteness and gamma band power, which may suggest that the right temporal lobe is somehow relevant to the abstractness/concreteness of the gesture/speech during multisensory integration. However, as we did not obtain the measure of familiarity and metaphoricity, we were unable to directly test whether these properties were relevant to the gamma oscillations as well as the activity in the RH. With regard to the lack of left IFG activation, this may originate from the stimulus difference between our study and previous studies that investigate predominantly linguistic metaphors: our stimuli were abstract sentences (e.g., *the presentation is on a high level*) but not as MP as genuine linguistic metaphors (e.g., *all lawyers are sharks*). Therefore, if metaphoricity is directly supported by the left IFG, then this region may not be activated with our experimental design. Therefore, further EEG studies that investigate the relationship between gamma oscillations and the role of familiarity, novelty, and metaphoricity of figurative co-speech gestures are clearly necessary.

Our findings can also be related to fMRI studies that examined multisensory integration during MP co-speech gesture processing using comparable video-material as in the current study (Kircher et al., 2009; Straube et al., 2011). For example, in Kircher et al. (2009), the authors used fMRI to compare the processing of MP co-speech gestures with both MP gestures without speech and MP speech events without any gestures. A conjunction of these two contrasts yielded increased activation in not only left fronto-temporal regions including the left IFG, but also in the right middle temporal gyrus. Similar right middle temporal gyrus activation for MP gestures was also reported by Straube et al. (2011). It has to be noted that, in this study, besides the bilateral integration effects for both MP and IC gestures, the authors also reported that the left IFG was specifically related to the semantic integration of MP vs. ICG. Thus, despite highly comparable paradigms and stimulus materials used, source localization of gamma effects and BOLD responses from fMRI point to different yet overlapping brain regions that are related to MP gesture integration. Nevertheless, our findings, together with previous fMRI studies, showed that the RH, especially the right middle temporal gyrus, is somehow relevant to the processing of MP co-speech gestures. Further M/EEG studies or combined EEG–fMRI studies are needed, to further specify the differential/common role of this region and the left IFG during the processing of MP vs. literal meanings in multisensory settings.

To summarize, for the first time, our study investigated the role of gamma oscillations for the integration of MP vs. IC gestures with respective sentence contexts. Our results extend the functional role of gamma oscillations during language processing, that is, gamma band oscillations are related not only to semantic integration/unification during sentence-level processing, but also to natural semantic integration/unification processes across modalities.

## Ethics Statement

This study was carried out in accordance with the recommendations of guidelines for human subjects, Ethics Committee at the Philipps University Marburg. The protocol was approved by the Ethics Committee at the Philipps University Marburg. All subjects gave written informed consent in accordance with the Declaration of Helsinki.

## Author Contributions

YH, AN, and BS conceptualized and designed the study. YH acquired, analyzed, and interpreted the data. YH, AN, MS, and BS wrote the manuscript.

## Conflict of Interest Statement

The authors declare that the research was conducted in the absence of any commercial or financial relationships that could be construed as a potential conflict of interest.
